# The effect of ovarian response parameters and the synergistic effect of assisted reproduction of poor ovarian response treated with platelet rich plasma: systematic review and meta-analysis

**DOI:** 10.1186/s12905-024-03101-3

**Published:** 2024-04-27

**Authors:** Wanjing Li, Jinbang Xu, Disi Deng

**Affiliations:** 1 Fujian Maternity and Child Health Hospital College of Clinical Medicine for Obstetrics & Gynecology and Pediatrics, Fujian Medical University, Fuzhou, P.R. China; 2 Hospital of Chengdu University of Traditional Chinese Medicine, Chengdu, P.R. China; 3 Chengdu University of Traditional Chinese Medicine, Chengdu, China

**Keywords:** Platelet rich plasma, Poor ovarian response, Infertility, Assisted reproduction, IVF-ET, Cell therapy

## Abstract

**Background:**

Poor ovarian response (POR) patients often encounter cycle cancellation and egg retrieval obstacles in assisted reproductive technology. Platelet rich plasma (PRP) ovarian injection is a potential treatment method, but the treatment methods are different, and the treatment results are controversial.

**Objective:**

This study adopts a systematic review and meta-analysis method based on clinical research to explore the efficacy and safety of PRP injection on POR.

**Method:**

The following databases were searched for research published before March 2023; Medline (via PubMed), Web of Science, Scopus, Cochrane Library, Embase, Cochrane Library, and China National Knowledge Infrastructure Database (CNKI). The literature was then screened by two independent researchers, who extracted the data and evaluated its quality. Research was selected according to the inclusion criteria, and its quality was evaluated according to the NOS standard Cohort study. The bias risk of the included study was assessed with STATE 14.0. RevMan 5.3 software was used for meta-analysis.

**Main results:**

Ten studies were included in the analysis, including 7 prospective cohort studies and 3 retrospective studies involving 836 patients. The results showed that after PRP treatment, follicle stimulating hormone (FSH) significantly decreased and anti-Mueller hormone (AMH) and luteinizing hormone (LH) significantly increased in POR patients, but estradiol did not change significantly; The number of antral follicles increased, and the number of obtaining eggs and mature oocytes significantly increased; The number of Metaphase type II oocytes, 2PN and high-quality embryos, and cleavage stage embryos significantly increased. In addition, the patient cycle cancellation rates significantly decreased. The rate of natural pregnancy assisted reproductive pregnancy and live birth increased significantly. Four reports made it clear that no adverse reactions were observed.

**Conclusion:**

PRP may have the potential to improve pre-assisted reproductive indicators in POR patients, increase the success rate of in vitro fertilization-embryo transfer (IVF-ET) in POR patients, and improve embryo quality, and may be beneficial to the pregnancy outcome. There is no obvious potential risk in this study, but further clinical support is still needed.

**Supplementary Information:**

The online version contains supplementary material available at 10.1186/s12905-024-03101-3.

## Introduction

Poor ovarian response (POR) refers to decreased reserve and poor response of the ovary to exogenous Gonadotropin (Gn) [1], and the patients usually suffer defects in live birth rate (reported average cumulative live birth rate of 56%, while non-POR patients reached at least 70%) and a higher rate of treatment discontinuation [2, 3]. The research suggests that the incidence rate of POR ranges from 3 to 10%. The factors leading to POR were related to genetic factors, living habits, chronic diseases, surgery or chemotherapy, autoimmune diseases, age, etc. With the introduction of the Patient-Oriented Strategies Encompassing IndividualizeD Oocyte Number (POSEIDON) standard (proposed in 2016), the Assisted Reproductive Therapy (ART) of POR has entered an individualized era guided by the number of oocytes [4]. The core of successful ART depends on obtaining enough oocytes, so it must face the challenges of POR (such as lack of oocytes obtained, low fertilization and high-quality embryos rate, high rate of cycle cancellation, low clinical pregnancy rate, low live birth rate, etc.). Reduced ovarian reserve was highly correlated with POR, and it was a vital limiting factor for the success of any infertility treatment method, which is characterized by a decrease in the number and quality of oocytes [these attributes being typically assessed through Anti-Müllerian Hormone (AMH) levels and the count of antral follicles], leading to low pregnancy and high miscarriage rates [5].

Patients with POR were usually recommended supportive therapy, which may include the growth hormone, testosterone or dehydroepiandrosterone, antioxidants, vitamins or Coenzyme Q10, etc. [6]. Previous meta-analysis compared these supportive therapies; among them, the cycle cancellation rate of Coenzyme Q10 was the most significantly reduced, and dehydroepiandrosterone and growth hormone have the most oocytes [7], however, these are only the results of a mutual comparison. Virtually, the clinical efficacy of these supportive therapies was often unstable. In recent years, local injection of platelet rich plasma (PRP) has come to the forefront as a treatment for POR, and current data shows its promising adjuvant therapeutic effects.

PRP is a liquid fraction of processed autologous peripheral blood with platelet concentrations higher than baseline, and is a cell therapy method. The mechanism of cell therapy is to induce the production of cytokines [8], PRP could release many bioactive factors and adhesion proteins at the injection site to repair tissue, and stimulate the cell function through the regulation of these substances on endocrine, paracrine signaling and autocrine mechanisms [9]. PRP was widely used in dermatology, cardiac surgery, plastic surgery, pain department, spinal disease and sports medicine [10], such as accelerated wound healing [11] and tendon regeneration [12]. In gynecology, PRP was used to treat intractable plasma cell vulvitis and recurrent implantation difficulties caused by thin endometrium [13, 14]. In recent years, scholars have attempted to use PRP in patients with POR and decreased ovarian reserve, and seemed to have achieved encouraging results [15], unfortunately, there was a lack of large-scale clinical research to support this, so this treatment method is still controversial in clinical practice at the time of writing [16]. There were two main controversies: A. Could PRP improve pregnancy outcomes in POR patients? B. Was the clinical application of PRP safe? In recent years, scholars have conducted meta-analysis on the study of intrauterine injection of PRP in patients with repeated embryo transplantation failures, and confirmed that it could increase embryo implantation rate and reduce the miscarriage rate [17]. Nevertheless, there was not a meta-analysis to answer the above controversy regarding the treatment of POR by intraovarian injection of PRP. Therefore, this study attempted to answer the above questions through systematic review and meta-analysis.

## Methods

### Protocol registration and reporting format

This system review and meta-analysis were reported based on The Preferred Reporting Items for Systematic Reviews and Meta-Analyses (PRISMA-NMA) [18]. The self-assessment results were shown in Table S1. The study has been registered and reviewed with the PROSPERO (ID: CRD42023400112). No ethical approval or patient consent was required.

### Focus question

Can PRP intraovarian injection improve the pregnancy outcome in POR patients? Is PRP clinical application safe?

### Search strategies

The following databases were searched for research published before February 2023; Medline (via PubMed), Web of Science, Scopus, Cochrane Library, Embase, Cochrane Library, and China National Knowledge Infrastructure Database (CNKI). The following medical keywords [MeSH] and Boolean operators were applied: “Platelet-Rich Plasma”, “Plasma, Platelet-Rich”, “Platelet Rich Plasma”, “Gonadotropin-Resistant Ovary Syndrome”, “Gonadotropin Resistant Ovary Syndrome”, “Resistant Ovary Syndrome”, “Primary Ovarian Insufficiency”, “Menopause, Premature”, “Premature Menopause”, “Ovarian Insufficiency, Primary”, “Ovarian Failure, Premature”, “Premature Ovarian Failure”. Due to the fact that decreased ovarian reserve (including premature ovarian failure and premature ovarian insufficiency) was the important inducer of POR, we expanded keywords to include POF for better literature access. There were no language restrictions, and the detailed search strategies were shown in the Table S2.

### Inclusion criteria

The inclusion criteria were built around the PICOS standard:

1. Participants: The included subjects were POR patients who received IVF-ET.
2. Intervention measures: PRP intraovarian injection.
3. Comparison: Not receiving PRP treatment.
4. Outcome: The serum concentration of estradiol, Follicle Stimulating Hormone (FSH) and luteinizing hormone (LH). Endometrial thickness and the count of mature oocytes, antral follicles, oocytes retrieved, metaphase type II (MII) oocytes and excellent embryos, duration of stimulation, Gn and estradiol trigger dose, the count of 2 Prokaryotic Embryos (2PN) and embryos on Day 5, pregnancy and cancellation rate.
5. Study design: Randomized controlled trial (RCT), prospective cohort study, retrospective study.

### Exclusion criteria

The exclusion criteria were as follows:

1. Experimental group or control group combined with other treatment methods.
2. Unable to extract outcome indicators or unable to obtain full text literature.
3. Experimental research, experience summary, case report.
4. Excluded the repeated publication of data and the research without diagnostic criteria.

### Literature screening and data extraction

The selected literature was imported into Excel 2016 for the purposes of management and the deletion of duplicate entries. After independently screening the literature to determine whether it met the inclusion criteria, the two researchers read the abstract and full text to determine whether they met the inclusion criteria. The data extraction content includes publication and patient information, intervention and control measures, and outcome indicators.

### Quality evaluation

The cohort studies and retrospective studies adopted the Newcastle–Ottawa Quality Assessment Scale (NOS) standard. Risk bias was evaluated using EGGER of STATA 14.0, with *P* > 0.05 indicating no significant bias risk. Any differences have been resolved through discussions with senior researchers.

### Statistical analyses

RevMan 5.3 was used for meta-analysis. The dichotomous and continuous variable used Odds Ratio (OR) and Mean Difference (MD) measurement classification effect respectively. OR and MD were calculated using a 95% confidence interval (CI). In terms of heterogeneity, the statistical values of 5%, 50%, and 75% of I^2^ represent mild, moderate, and high heterogeneity, respectively, and were used to measure the presence of heterogeneity. STATA 14.0 was used for bias assessment.

## Results

### Search results and research selection

**Fig. 1:**
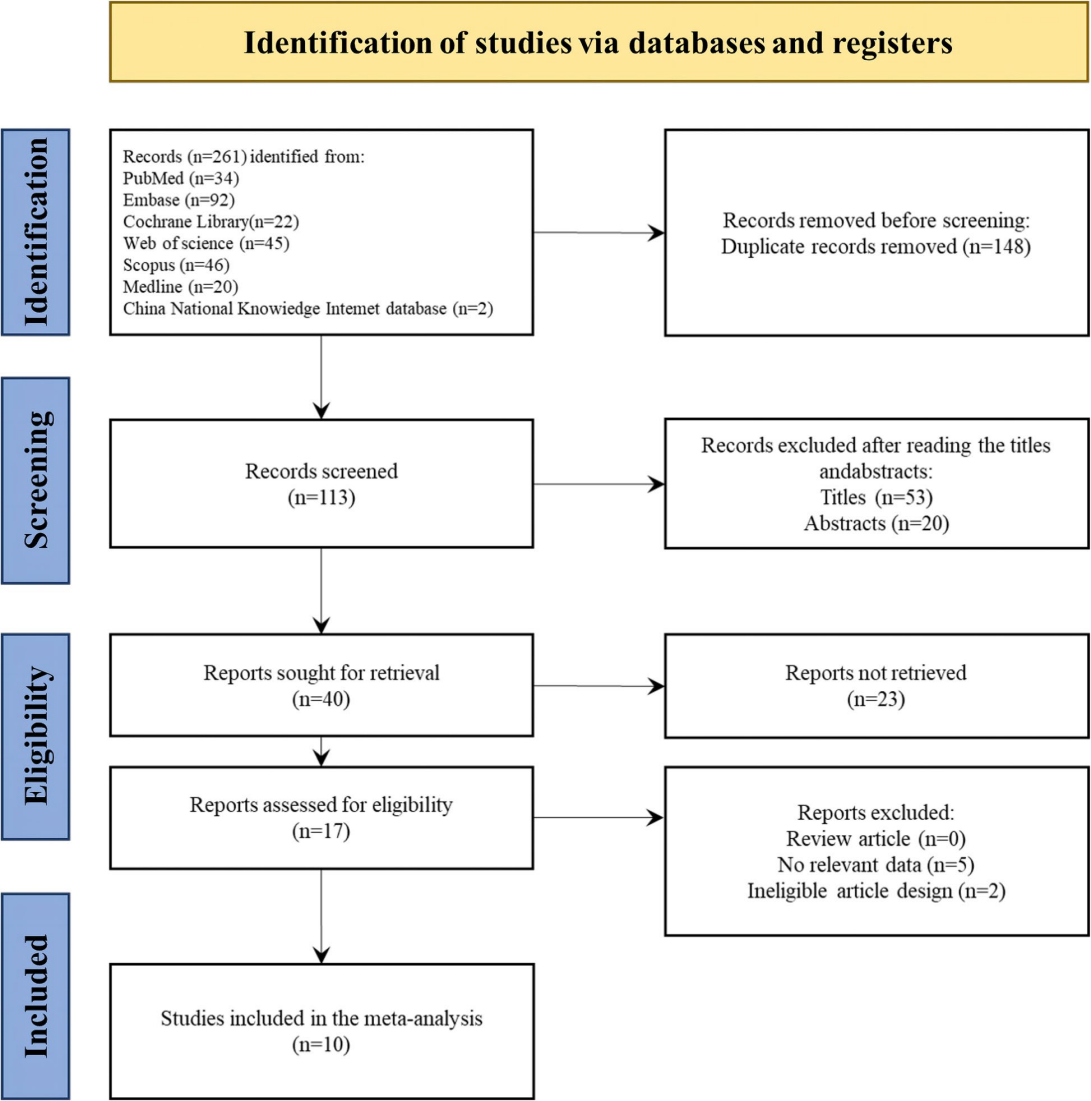
Retrieval flowchart for meta-analysis

As shown in the search flowchart in Fig. 1, the search strategy obtained 261 potentially relevant articles. According to the inclusion criteria, 40 articles were identified for further full-text evaluation. We included 10 studies for analysis, including 7 prospective cohort studies and 3 retrospective studies, which involved 836 patients. Table S3 showed the characteristics of the studies included in the meta-analysis. These studies were all conducted post-2017, indicating that PRP intraovarian injection is a novel clinical treatment strategy. The wide distribution of published regions indicated that the method had initially received clinical recognition.

### Result of quality evaluation

Table S4 showed the quality assessment results of the included studies. The results showed that the quality of the included literatures were relatively high (NOS: 4 ± 0.47), and the factors affected the quality of literatures were concentrated in the sample size of the included studies (most of them less than 200 people).

### Results of PRP injection methods and ART selection

Table S5 showed the information of type of ART, excretion promotion scheme and PRP injection dose, mode and treatment opportunity included in the articles. The results showed that seven studies used ovulation induction, including one light stimulation ovulation induction, two combined with intracytoplasmic sperm injection, and two non-ovulation induction. The injection dose of PRP ranged from 1.5 to 4 ml, and two studies did not report the injection dose. Among them, four studies injected 2 ml, two studies injected 4 ml, and 1 study injected 1.5 ml. Three studies identified unilateral ovarian injection, six studies identified bilateral ovarian injection, and one study reported at least unilateral ovarian injection. The frequency of treatments ranged from 1 to 2, but the interval time was significantly different (1–3 months). Ovarian hormone levels were measured before the first PRP injection, at the first menstrual baseline after treatment, and after HCG injection, respectively. The long-term efficacy was observed at 3 and 6 months after PRP treatment.

### Meta analysis of POR treatment results

#### Meta analysis of ovarian sex hormones and ovarian reserve indicators

Figure S1 showed a forest map of estradiol, FSH, LH, and AMH in POR patients. The results showed that PRP did not significantly affect the levels of estradiol and LH in POR women (-37.81 [-92.78, 17.15], 0.95 [-0.68, 2.59], respectively), but patients treated with PRP had a significant decrease in FSH (1.52 [0.11, 2.94]) and a significant increase in AMH (-0.18 [-0.32, -0.04]). We analyzed the heterogeneity of the results one by one, and the results of estradiol, FSH, and AMH were consistent with the above (0.11 [-5.95,6.17], 1.87 [1.20, 2.55], -0.18 [-0.22, -0.14], respectively). However, the level of LH significantly increased after removing the high heterogeneity documents (-0.14 [-0.25, -0.02]).

#### Meta analysis of ovarian reserve and IVF-ET circulation indicators

Figure S2 showed the forest map of follicular development status and circulation indicators in POR patients. The results showed that after PRP treatment, the count of antral follicles significantly increased (-1.47 [-2.18, -0.77]), the stimulation time and the dosage of Gn had no significant difference (0.01 [-0.41, 0.42], 62.19[-179.60 55.22], respectively), the dosage of estradiol for HCG trigger significantly increased (-560.03 [-1058.41, -61.65]), the count of oocytes retrieved and mature oocytes obtained significantly increased (-1.16 [-1.45, -0.87], -1.37 [-1.45, -1.29], respectively). After in vitro culture, the number of MII type oocytes increased (-1.10 [-1.69, -0.51]), the count of 2PN significantly increased (-0.93 [-1.52, -0.35]), the count of high-quality embryos significantly increased (-1.04 [-1.87, -0.20]), and the number of embryos in the cleavage stage significantly increased (-0.86 [-1.18, -0.53]). Additionally, the patient cycle cancellation rate significantly decreased (2.25 [1.30, 3.89]).

#### Meta analysis of pregnancy rate and pregnancy outcome

Figure S3 showed the forest map of pregnancy rate and pregnancy outcome. The results showed that after PRP treatment, the natural and ART pregnancy rate significantly increased (0.06 [0.01, 0.20] and 0.02 [0.01, 0.06], respectively). The live birth rate was significantly higher in cases of pregnancy (0.02 [0.01, 0.10]).

#### Meta analysis of endometrial thickness

There were no significant differences in endometrial thickness (0.03 [-0.30,0.36]).

### Result of safety

As shown in Table S5, among the 10 included studies, 4 clearly reported there were not complications caused by PRP ovarian injection (including infection, bleeding, fever, pelvic inflammation, etc.), and one specifically pointed out that the safety of operation by experts could be guaranteed.

### Publication bias results

EGGER results showed no significant publication bias in the following results: estradiol (*P* = 0.173), FSH (*P* = 0.07), AMH (*P* = 0.192), LH (*P* = 0.43), the count of antral follicles (*P* = 0.799), stimulation time (*P* = 0.934), and count of mature oocytes obtained (*P* = 0.515). The count of MII type oocytes (*P* = 0.346), 2PN (*P* = 0.709), high-quality embryos (*P* = 0.170), and cleavage embryos (*P* = 0.882).

## Discussion

### Preparation method and mechanism of PRP

The general process of PRP preparation included collecting whole blood, preliminary centrifugation to separate red blood cells, followed by further centrifugation to concentrate platelets, and finally adding platelet agonists to activate platelets, such as calcium containing additives [19]. It contains 5 to 10 times the high concentration of growth factors released by activated platelets. The results of this study were consistent with those reported in the literature. Most of the patients were treated with 2 ml multi-point injection into bilateral ovaries. Because the follicular development was periodic, the therapeutic effect usually took effect 1–2 months after PRP injection, and it lapsed after about 6 months. A rule of the basis for the selection of injection dose was not found, but the result reaction requires at least 2 ml injection to have a therapeutic effect. The interval between two injections of PRP should be at least one menstrual cycle, but there was no unified treatment standard at present. The observation nodes reported in different literatures based on the research objectives have obvious differences. The detection of ovarian endocrine function was mostly concentrated in the baseline period of the first menstrual cycle after PRP registration, while the detection of assisted reproductive cycle parameters was mostly concentrated after the trigger day.

The efficiency of PRP mainly depends on its α particle content [20], including transforming growth factor- β (TGF-β), fibroblast growth factor, insulin-like growth factor 1/2, vascular endothelial growth factor(VEGF) and epidermal growth factor [21]. Moreover, GDF-9, which was closely relevant to the maturation potential of oocytes, was also present in PRP [22]. They attached to cell membrane receptors, mediate important biological effects, and control cell growth, proliferation, and differentiation by regulating intracellular signaling pathways. Different from hormones, these growth factors showed rather limited activity and only exert local effects when very close to their release sites, including mitosis, angiogenesis, chemotaxis and the formation of extracellular matrix, and even control the release of other growth factors [23]. It was reported that PRP had the ability to reduce oxidative stress and inflammation through VEGF signaling pathway, and its high content of growth factors could also preserve the function and structure of ovarian torsion during conservative ovarian surgery [24]. This might be related to multiple growth factors in PRP regulating the cell function, improving tissue microenvironment, and/or regulating tissue regeneration [23]. In addition, the failure of organizational function involves coordinated tissue remodeling and complex structural regeneration [25]. Current research recognizes that the immune system plays an crucial supporting role in ovarian function, especially in follicular development. Obstacles to immune regulatory function in the ovaries were believed in the cause of ovarian dysfunction [23]. High levels of inflammatory cytokines (such as IL-1, IL-6, IL-8), growth factors (such as TGF-β, hepatocyte growth factor, VEGF, platelet derived growth factor), matrix metallo proteinase and cathepsin are highly likely to be related to tissue aging [25], and the regulation of these phenotypes was mostly in the direct or indirect regulation category of PRP. In addition, when PRP was used to intervene in aging bone marrow stem cells of elderly mice, the results showed that it could restore various aging stem cell functions [26], there were reports of combining stem cell transplantation with PRP in the treatment of ovarian failure, confirming its dose-dependent assistance in follicle regeneration [27], PRP has the potential to combine with stem cell transplantation technology to restore ovarian function. Finally, in vitro studies have confirmed that PRP could stimulate granulosa cell proliferation and counteract inflammatory processes [28]. However, the results suggested that the improvement of ovarian function by PRP seems to aim at differentiation of precursor cell. In short, the mechanism of PRP was achieved by restoring local cellular function through various cytokines contained within it.

### Analysis of results

It was reported that the combined use of PRP and Gn in the ovarian full-dimensional subcortical could restore ovarian function [29], and the effect could be maintained for 2–6 months [30]. Consistent with the results of this study, it was found that the use of PRP could reduce FSH levels in POR patients, increase AMH levels, and increase the count of antral follicles,suggesting that PRP could increase ovarian reserve in POR patients. Although not all POR patients were accompanied by a decrease in ovarian reserve, high-quality and sufficient eggs were beneficial for POR patients. This regulation might be related to PRP concentration and the signaling pathway of ovarian angiogenesis [31]. The average age of patients included in this study was over 35 years old, and the grouping of age lacked detail, so the data cannot be more accurately subgrouped for analysis of the influence of age. However, Farimani et al. [32] made a subgroup analysis of influence of age and AMH level on the pregnancy outcome and ART parameters. The results showed that both of them greatly affected the therapeutic effect of PRP, especially the influence of age. This result was also reflected in the heterogeneity analysis of FSH, Farimani et al. [32] included a higher proportion of women aged 35 and above (50%), which undoubtedly led to the difficulty in reducing FSH levels, and led to high heterogeneity.It is consistent with the poor performance of PRP in diseases such as premature ovarian failure [33].

Secondly, by excluding highly heterogeneous literature, it was found that PRP could improve the LH level in POR patients, suggesting that the ovarian sensitivity to Gn has been improved. If the research results of Sfakianoudis were included in the analysis, the results are highly heterogeneous, and the research results no longer have statistical differences. Reviewing the literature, we believed that most of the patients included in the study have received controlled ovarian hyperstimulation therapy [20], and the use of Gn might have an impact on the adenohypophysis, leading to differences in results.

In addition, PRP had no significant regulation on estradiol levels in POR patients. Although PRP could locally enhance ovarian secretion function based on data, if it was not caused by decreased ovarian reserve function in POR patients, estradiol levels would not be significantly lower than normal levels. Previous studies have shown that if patients with ovarian reserve failure rely solely on PRP, the efficacy was poor and other treatment methods were needed [34]. But the efficacy may not be satisfactory. So far, the best way to treat infertility with ovarian reserve failure still was to receive donor eggs [35].

An increasing number of clinical adjuvant drugs were being considered for use in ART treatment, such as the growth hormone, aspirin, heparin, dehydroepiandrosterone, testosterone, antioxidants and hysteroscopy and these treatments were likely limited in effect and increased the total cost of treatment [36]. Injecting autologous PRP into the ovary demonstrates more accurate efficacy and lower treatment costs. Our analysis of the IVF-ET cycle indicators showed that PRP could increase the count of antral follicles in POR patients and obtain more oocyte retrieval and mature oocyte. PRP effectively replaced the beneficial effects of serum during in vitro oocyte maturation and maintained mitochondrial activity in mature oocytes [37]. However, the data showed that more estrogen was needed to trigger ovulation after the use of PRP, and it could not reduce the use of Gn and the stimulation time, which confirms the local effect of PRP. The mechanism of improving the ovulation efficiency might be the intra follicular mechanism, rather than improving the sensitivity of the ovary to Gn or the hypothalamus pituitary ovary axis. In addition, the quality of oocytes, division, and the number of high-quality embryos had significantly increased, and they could better enter the cleavage stage. The cancellation rate of the cycle has significantly decreased, and better reproductive outcomes have been achieved, which is consistent with the previously reported cases [38]. The analysis of pregnancy rate showed that some patients could have spontaneous pregnancy after injection of PRP, and the pregnancy rate combined with ART could be further improved, and that ART could significantly improve the live birth rate. Moreover, there was a case report that PRP successfully helped a relatively elderly woman (43 years old) with pregnancy and delivery who had failed ART ten times [39].

In addition, no significant difference in endometrial thickness among POR patients was found after ovarian injection of PRP, but the endometrial thickness of the POR patients included in the study was within the normal range before treatment. In addition, this result also suggested that multiple point injection of PRP might be necessary for patients with thin endometrium. It was reported that after intrauterine infusion of PRP, the endometrium expands and the pregnancy rate significantly increased, which could be used to treat POR patients with thin endometrium, and this reflected the enormous potential of PRP treatment [40].

### Security of PRP

PRP was autologous substance that could effectively avoid the common and serious threat of autoimmune reactions based on its special source [41]. So far, no serious side effects have been observed after PRP enters the reproductive organs. But mild complications such as local pain, irritation, erythema, and swelling around the injection site have been reported in other tissues. These complications have not been reported in the treatment of POR [42], and the studies included here did not report on this issue. Therefore, we could get an encouraging directional result, that is, the intra ovarian injection (< 4 ml) completed by professionals under ultrasound-guided or laparoscopic vision might be relatively safe, and age was not the influencing factor of safety without ovarian atrophy. However, scholars have many doubts about the safety of PRP, and it has been reported that PRP samples have been found to be positive for microbial growth. However, there was no conclusive evidence of association [43, 44]. In the field of reproduction, there have been no reports confirming abnormal ovarian responses or excessive stimulation effects of PRP [45]. However, PRP treatment might have unknown potential adverse effects [46], such as the harmful effects of high concentrations of hematopoietic cells on embryos [47]. In addition, stem cell transplantation was a potential treatment option for patients with ovarian reserve failure. Some research reported that the combination of stem cell transplantation and PRP might be more effective. However, stem-cell therapy was related to tumorigenesis, making the combination of PRP less safe than other methods.

Overall, the results of this study were encouraging that they confirm the synergistic effect of PRP in ART, which was effective and safe for improving indicators of low ovarian reserve before ART, as well as improving the quality and success rate of in vitro cultured embryos, consistent with previous studies [48], and achieving better pregnancy outcomes. It is worth noting that none of the studies have reported significant side effects. This was both an opportunity and a challenge for PRP, and future research should be cautious when investigating this.

## Innovation and limitations of this study

This study provided the first objective analysis of the therapeutic effect and safety of PRP on POR, supported by a large sample size. However, this research did not cover the data on the health assessment of live born fetuses, as well as the assessment of the follow-up fertility of patients with this treatment method. Moreover, most of the studies included in this study were prospective and retrospective studies, and large-scale RCT studies were not yet sufficient. It is hoped that the affirmation of the efficacy and safety of this study's results could promote the emergence of more clinical RCT studies.

## Conclusion

The application of PRP before the use of ART is beneficial the ovarian response of patients with POR, obtaining more antral follicles, improving the success rate of IVF-ET and leading to better reproductive outcomes, but there is a lack of standardized treatment. In addition, current studies have shown that PRP ovarian injection is safe. However, due to the lack of randomized controlled studies as support, the clinical application of PRP still needs to be treated with caution.

### Supplementary Information

**Supplementary Material 1.**

## Data Availability

Data is provided within the manuscript or supplementary information files.
