# Supplementary material for: The effect of ovarian response parameters and the synergistic effect of assisted reproduction of poor ovarian response treated with platelet rich plasma: systematic review and meta-analysis
Source: BMC Womens Health. 2024 Apr 27;24:263. doi: 10.1186/s12905-024-03101-3 (PMC11055225; doi:10.1186/s12905-024-03101-3)
Supplement: Supplementary file 1 — Supplementary Material 1. [file 12905_2024_3101_MOESM1_ESM.docx]

| A | 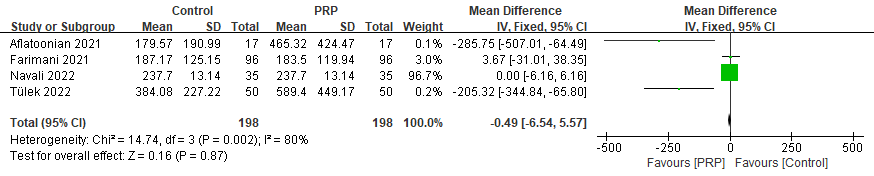 | 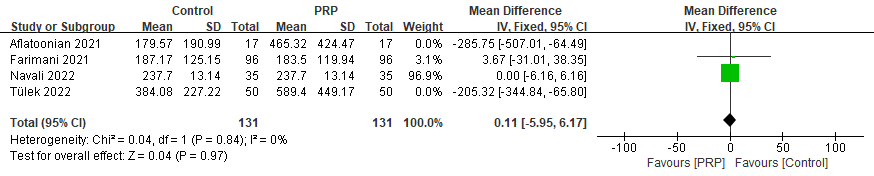 |
| --- | --- | --- |
| B | 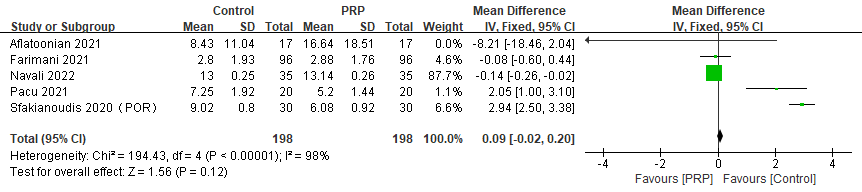 | 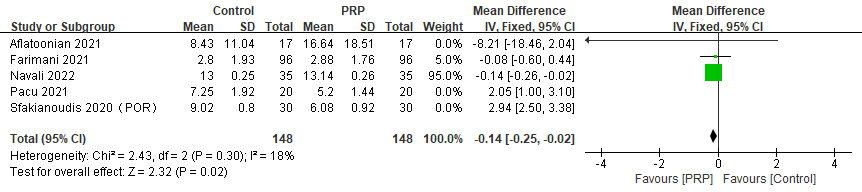 |
| C | 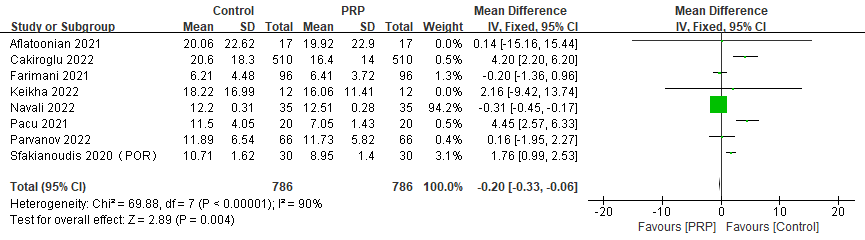 | 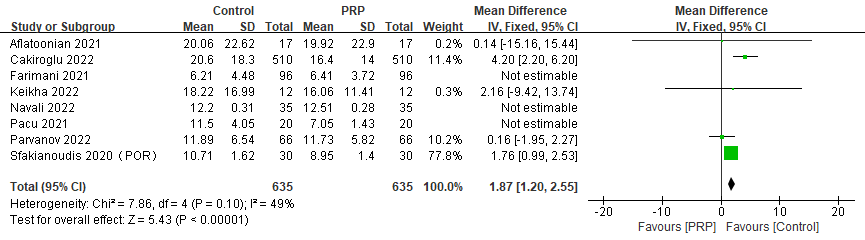 |
| D | 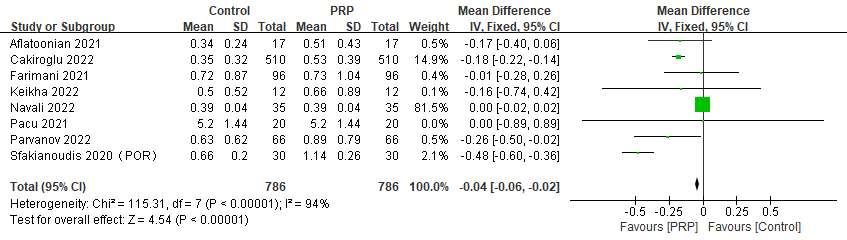 | 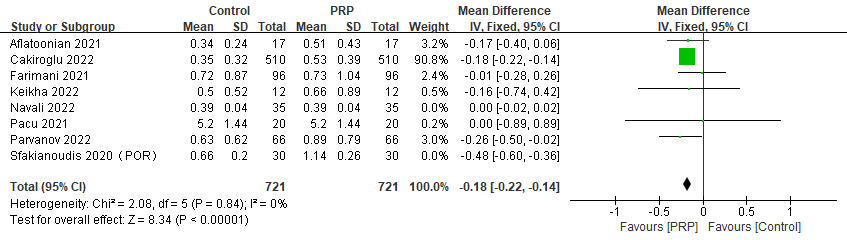 |

Figure S1. Forest plot of sex hormone of POR treated with PRP (A. E_2_ B. LH C. FSH D. AMH，The left side shows the results of unadjusted heterogeneity, and the right side shows the results of low heterogeneity)

| A | 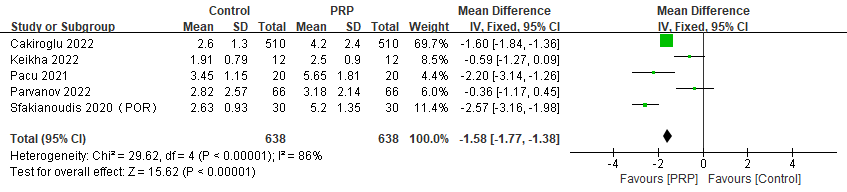 | 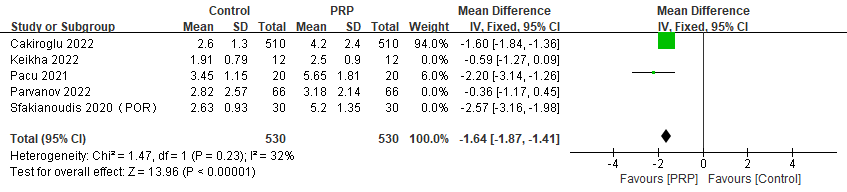 |
| --- | --- | --- |
| B | 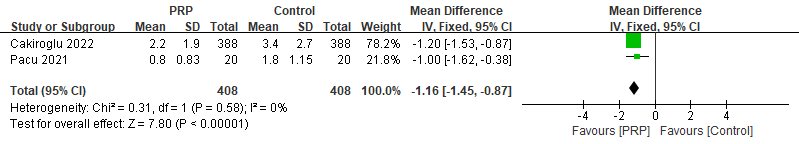 |  |
| C | 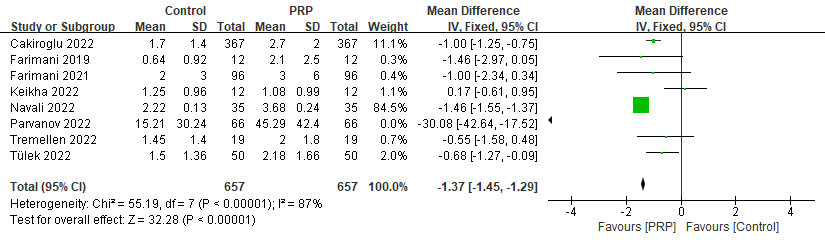 | 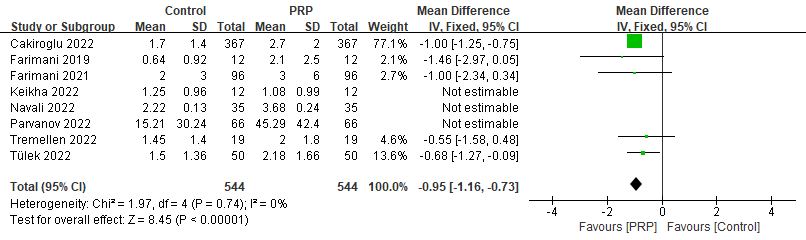 |
| D | 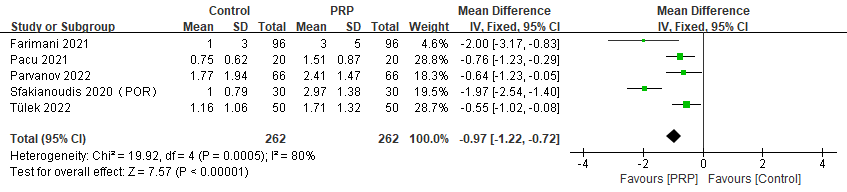 | 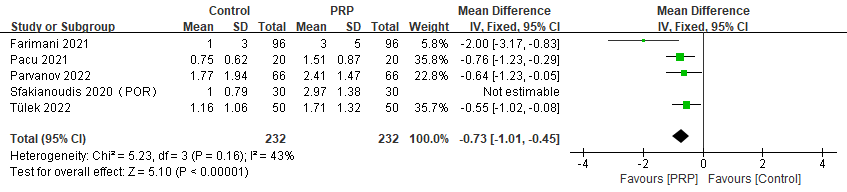 |
| E | 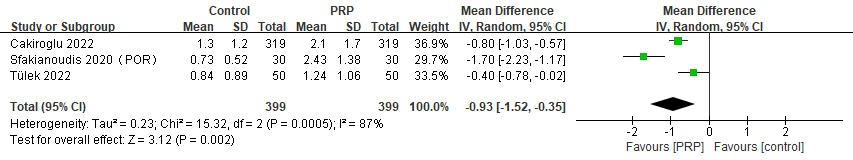 |  |
| F | 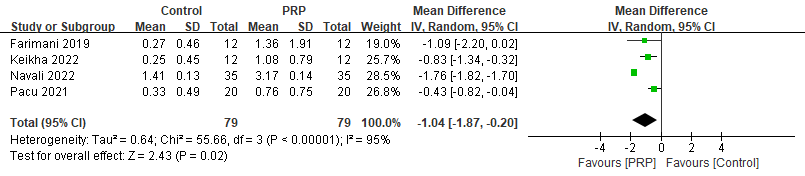 | 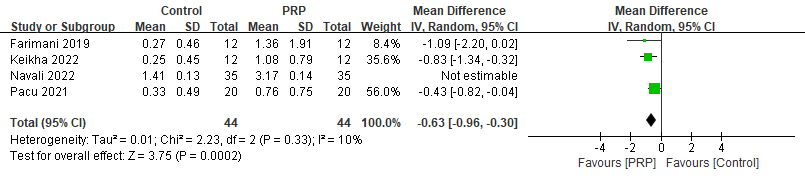 |
| G | 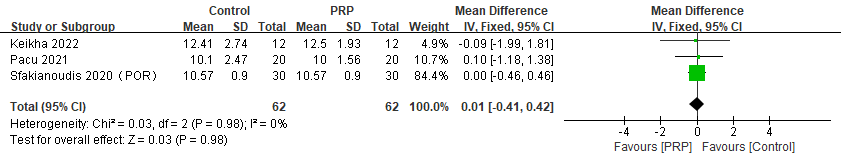 |  |
| H | 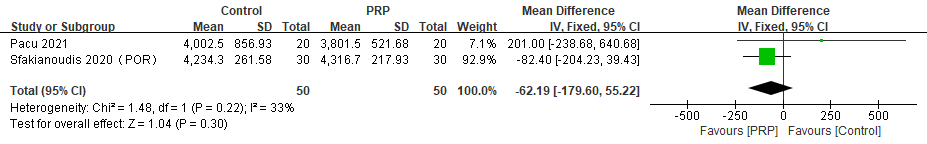 |  |
| I | 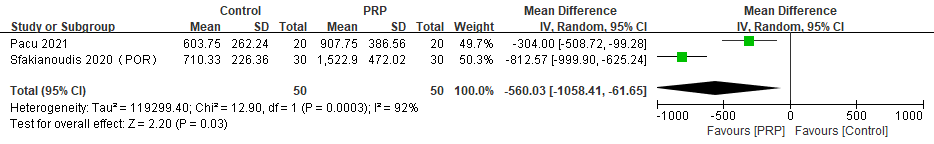 |  |
| J | 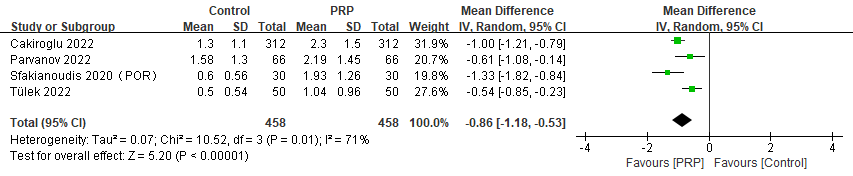 | 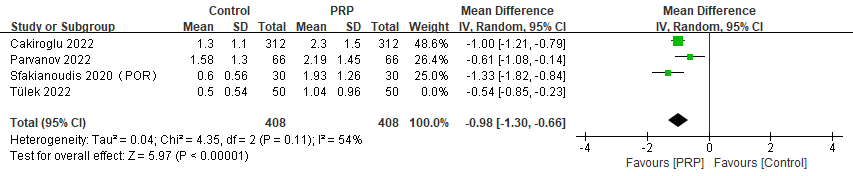 |
| K | 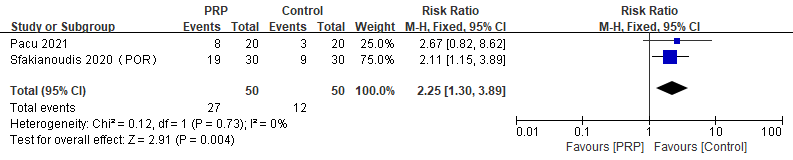 |  |

Figure S2. Forest plot of follicular development status and circulation indicators (A. antral follicle count B. Obtaining eggs count C. Mature oocyte count D. Metaphase type II oocytes count E. 2PN count F. High quality embryo count G. Gonadotropin stimulation time H. Gonadotropin usage I. Estradiol triggering amount J. Embryo count during cleavage stage K. Cycle cancellation rate. The left side shows the results of unadjusted heterogeneity, and the right side shows the results of low heterogeneity.)

| A | 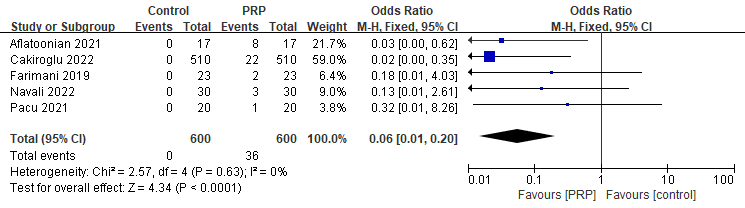 | B | 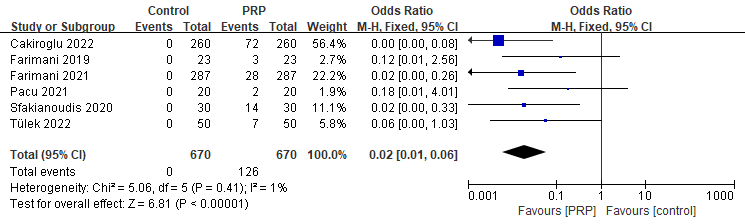 |
| --- | --- | --- | --- |
| C | 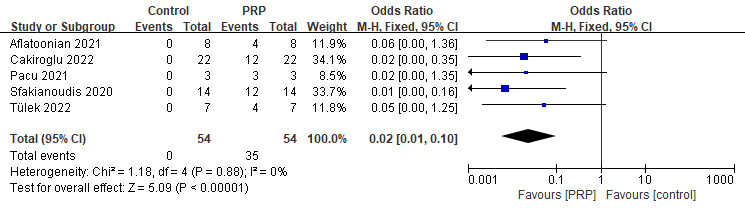 |  |  |

Figure S3. Forest plot of pregnancy rate and pregnancy outcome. (A. natural pregnancy rate. B.ART pregnancy rate. C. live birth rate.)

Table S1. PRISMA checklist

| **Section/topic** | **#** | **Checklist item** | **Reported on page #** |
| --- | --- | --- | --- |
| **TITLE** | | |  |
| Title | 1 | Identify the report as a systematic review, meta-analysis, or both. | 1 |
| **ABSTRACT** | | |  |
| Structured summary | 2 | Provide a structured summary including, as applicable: background; objectives; data sources; study eligibility criteria, participants, and interventions; study appraisal and synthesis methods; results; limitations; conclusions and implications of key findings; systematic review registration number. | 1 |
| **INTRODUCTION** | | |  |
| Rationale | 3 | Describe the rationale for the review in the context of what is already known. | 2 |
| Objectives | 4 | Provide an explicit statement of questions being addressed with reference to participants, interventions, comparisons, outcomes, and study design (PICOS). | 3 |
| **METHODS** | | |  |
| Protocol and registration | 5 | Indicate if a review protocol exists, if and where it can be accessed (e.g., Web address), and, if available, provide registration information including registration number. | 2 |
| Eligibility criteria | 6 | Specify study characteristics (e.g., PICOS, length of follow-up) and report characteristics (e.g., years considered, language, publication status) used as criteria for eligibility, giving rationale. | 3 |
| Information sources | 7 | Describe all information sources (e.g., databases with dates of coverage, contact with study authors to identify additional studies) in the search and date last searched. | 2 |
| Search | 8 | Present full electronic search strategy for at least one database, including any limits used, such that it could be repeated. | Table S2 |
| Study selection | 9 | State the process for selecting studies (i.e., screening, eligibility, included in systematic review, and, if applicable, included in the meta-analysis). | 4 |
| Data collection process | 10 | Describe method of data extraction from reports (e.g., piloted forms, independently, in duplicate) and any processes for obtaining and confirming data from investigators. | 3 |
| Data items | 11 | List and define all variables for which data were sought (e.g., PICOS, funding sources) and any assumptions and simplifications made. | NA |
| Risk of bias in individual studies | 12 | Describe methods used for assessing risk of bias of individual studies (including specification of whether this was done at the study or outcome level), and how this information is to be used in any data synthesis. | 3 |
| Summary measures | 13 | State the principal summary measures (e.g., risk ratio, difference in means). | 3 |
| Synthesis of results | 14 | Describe the methods of handling data and combining results of studies, if done, including measures of consistency (e.g., I^2^) for each meta-analysis. | 3 |
| Risk of bias across studies | 15 | Specify any assessment of risk of bias that may affect the cumulative evidence (e.g., publication bias, selective reporting within studies). | 3-4 |
| Additional analyses | 16 | Describe methods of additional analyses (e.g., sensitivity or subgroup analyses, meta-regression), if done, indicating which were pre-specified. | NA |
| **RESULTS** | | | |
| Study selection | 17 | Give numbers of studies screened, assessed for eligibility, and included in the review, with reasons for exclusions at each stage, ideally with a flow diagram. | 4  Fig. 1 |
| Study characteristics | 18 | For each study, present characteristics for which data were extracted (e.g., study size, PICOS, follow-up period) and provide the citations. | Table S4 |
| Risk of bias within studies | 19 | Present data on risk of bias of each study and, if available, any outcome level assessment (see item 12). | Table S3 |
| Results of individual studies | 20 | For all outcomes considered (benefits or harms), present, for each study: (a) simple summary data for each intervention group (b) effect estimates and confidence intervals, ideally with a forest plot. | 4-5  Figure S1 |
| Synthesis of results | 21 | Present results of each meta-analysis done, including confidence intervals and measures of consistency. | 4-5 |
| Risk of bias across studies | 22 | Present results of any assessment of risk of bias across studies (see Item 15). | 5 |
| Additional analysis | 23 | Give results of additional analyses, if done (e.g., sensitivity or subgroup analyses, meta-regression [see Item 16]). | NA |
| **DISCUSSION** | | | |
| Summary of evidence | 24 | Summarize the main findings including the strength of evidence for each main outcome; consider their relevance to key groups (e.g., healthcare providers, users, and policy makers). | 2 |
| Limitations | 25 | Discuss limitations at study and outcome level (e.g., risk of bias), and at review-level (e.g., incomplete retrieval of identified research, reporting bias). | 8 |
| Conclusions | 26 | Provide a general interpretation of the results in the context of other evidence, and implications for future research. | 7 |
| **FUNDING** |  |  |  |
| Funding | 27 | Describe sources of funding for the systematic review and other support (e.g., supply of data); role of funders for the systematic review. | 8 |

*From:*  Moher D, Liberati A, Tetzlaff J, Altman DG, The PRISMA Group (2009). Preferred Reporting Items for Systematic Reviews and Meta-Analyses: The PRISMA Statement. PLoS Med 6(7): e1000097. doi:10.1371/journal.pmed1000097

Table S2. Retrieval strategies for each database

| Database | Full search strategy |
| --- | --- |
| Pubmed(33) | #1 "Primary Ovarian Insufficiency" OR "Menopause, Premature" OR "Premature Menopause" OR "Ovarian Insufficiency, Primary" OR "Ovarian Failure, Premature" OR "Premature Ovarian Failure" OR "Gonadotropin-Resistant Ovary Syndrome" OR "Gonadotropin Resistant Ovary Syndrome" OR "Resistant Ovary Syndrome" OR "Hypergonadotropic Ovarian Failure, X-Linked" OR "Hypergonadotropic Ovarian Failure, X Linked" OR "X-Linked Hypergonadotropic Ovarian Failure" OR "X Linked Hypergonadotropic Ovarian Failure" OR "Premature Ovarian Failure, X-Linked" OR "Premature Ovarian Failure, X Linked" OR "Fragile X-Associated Primary Ovarian Insufficiency" OR "Fragile X Associated Primary Ovarian Insufficiency" OR "Fragile X Premature Ovarian Failure" OR "FMR1-Related Primary Ovarian Insufficiency" OR "FMR1 Related Primary Ovarian Insufficiency" OR "Primary Ovarian Insufficiency, Fragile X-Associated" OR "Primary Ovarian Insufficiency, Fragile X Associated" OR "Premature Ovarian Failure 1" OR "poor ovarian response"[MeSH Terms]  #2 "Platelet-Rich Plasma" OR "Plasma, Platelet-Rich" OR "Platelet Rich Plasma"[MeSH Terms]  #3 #1 AND #2 |
| Web of Science(45) | (TS=(“Primary Ovarian Insufficiency” OR “Menopause, Premature” OR “Premature Menopause” OR “Ovarian Insufficiency, Primary” OR “Ovarian Failure, Premature” OR “Premature Ovarian Failure” OR “Gonadotropin-Resistant Ovary Syndrome” OR “Gonadotropin Resistant Ovary Syndrome” OR “Resistant Ovary Syndrome” OR “Hypergonadotropic Ovarian Failure, X-Linked” OR “Hypergonadotropic Ovarian Failure, X Linked” OR “X-Linked Hypergonadotropic Ovarian Failure” OR “X Linked Hypergonadotropic Ovarian Failure” OR “Premature Ovarian Failure, X-Linked” OR “Premature Ovarian Failure, X Linked” OR “Fragile X-Associated Primary Ovarian Insufficiency” OR “Fragile X Associated Primary Ovarian Insufficiency” OR “Fragile X Premature Ovarian Failure” OR “FMR1-Related Primary Ovarian Insufficiency” OR “FMR1 Related Primary Ovarian Insufficiency” OR “Primary Ovarian Insufficiency, Fragile X-Associated” OR “Primary Ovarian Insufficiency, Fragile X Associated” OR “Premature Ovarian Failure 1” OR “poor ovarian response”)) AND TS=(“Platelet-Rich Plasma” OR “Plasma, Platelet-Rich” OR “Platelet Rich Plasma”) |
| EMBASE(92) | #1 'primary ovarian insufficiency'/mj OR 'menopause, premature'/mj OR 'premature menopause'/mj OR 'ovarian insufficiency, primary' OR 'ovarian failure, premature'/mj OR 'premature ovarian failure'/mj OR 'gonadotropin-resistant ovary syndrome' OR 'gonadotropin resistant ovary syndrome' OR 'resistant ovary syndrome'/mj OR 'hypergonadotropic ovarian failure, x-linked' OR 'hypergonadotropic ovarian failure, x linked' OR 'x-linked hypergonadotropic ovarian failure' OR 'x linked hypergonadotropic ovarian failure' OR 'premature ovarian failure, x-linked' OR 'premature ovarian failure, x linked' OR 'fragile x-associated primary ovarian insufficiency' OR 'fragile x associated primary ovarian insufficiency'/mj OR 'fragile x premature ovarian failure' OR 'fmr1-related primary ovarian insufficiency' OR 'fmr1 related primary ovarian insufficiency' OR 'primary ovarian insufficiency, fragile x-associated' OR 'primary ovarian insufficiency, fragile x associated' OR 'premature ovarian failure 1' OR 'poor ovarian response'/mj  #2 'platelet-rich plasma'/mj OR 'plasma, platelet-rich' OR 'platelet rich plasma'/mj  #3 #1 AND #2 |
| Medline(20) | #1 “Primary Ovarian Insufficiency” OR “Menopause, Premature” OR “Premature Menopause” OR “Ovarian Insufficiency, Primary” OR “Ovarian Failure, Premature” OR “Premature Ovarian Failure” OR “Gonadotropin-Resistant Ovary Syndrome” OR “Gonadotropin Resistant Ovary Syndrome” OR “Resistant Ovary Syndrome” OR “Hypergonadotropic Ovarian Failure, X-Linked” OR “Hypergonadotropic Ovarian Failure, X Linked” OR “X-Linked Hypergonadotropic Ovarian Failure” OR “X Linked Hypergonadotropic Ovarian Failure” OR “Premature Ovarian Failure, X-Linked” OR “Premature Ovarian Failure, X Linked” OR “Fragile X-Associated Primary Ovarian Insufficiency” OR “Fragile X Associated Primary Ovarian Insufficiency” OR “Fragile X Premature Ovarian Failure” OR “FMR1-Related Primary Ovarian Insufficiency” OR “FMR1 Related Primary Ovarian Insufficiency” OR “Primary Ovarian Insufficiency, Fragile X-Associated” OR “Primary Ovarian Insufficiency, Fragile X Associated” OR “Premature Ovarian Failure 1” OR “poor ovarian response”  #2 "Platelet-Rich Plasma" OR "Plasma, Platelet-Rich" OR "Platelet Rich Plasma"  #3 #1 AND #2 |
| Scopus(46) | TITLE-ABS-KEY(“Primary Ovarian Insufficiency” OR “Menopause, Premature” OR “Premature Menopause” OR “Ovarian Insufficiency, Primary” OR “Ovarian Failure, Premature” OR “Premature Ovarian Failure” OR “Gonadotropin-Resistant Ovary Syndrome” OR “Gonadotropin Resistant Ovary Syndrome” OR “Resistant Ovary Syndrome” OR “Hypergonadotropic Ovarian Failure, X-Linked” OR “Hypergonadotropic Ovarian Failure, X Linked” OR “X-Linked Hypergonadotropic Ovarian Failure” OR “X Linked Hypergonadotropic Ovarian Failure” OR “Premature Ovarian Failure, X-Linked” OR “Premature Ovarian Failure, X Linked” OR “Fragile X-Associated Primary Ovarian Insufficiency” OR “Fragile X Associated Primary Ovarian Insufficiency” OR “Fragile X Premature Ovarian Failure” OR “FMR1-Related Primary Ovarian Insufficiency” OR “FMR1 Related Primary Ovarian Insufficiency” OR “Primary Ovarian Insufficiency, Fragile X-Associated” OR “Primary Ovarian Insufficiency, Fragile X Associated” OR “Premature Ovarian Failure 1” OR “poor ovarian response”) AND TETLE-ABS-KEY(“Platelet-Rich Plasma” OR “Plasma, Platelet-Rich” OR “Platelet Rich Plasma”) |
| Cochrane Library(22) | #1 (“Primary Ovarian Insufficiency” OR “Menopause, Premature” OR “Premature Menopause” OR “Ovarian Insufficiency, Primary” OR “Ovarian Failure, Premature” OR “Premature Ovarian Failure” OR “Gonadotropin Resistant Ovary Syndrome” OR “Gonadotropin Resistant Ovary Syndrome” OR “Resistant Ovary Syndrome” OR “Hypergonadotropic Ovarian Failure, X Linked” OR “Hypergonadotropic Ovarian Failure, X Linked” OR “X Linked Hypergonadotropic Ovarian Failure” OR “X Linked Hypergonadotropic Ovarian Failure” OR “Premature Ovarian Failure, X Linked” OR “Premature Ovarian Failure, X Linked” OR “Fragile X Associated Primary Ovarian Insufficiency” OR “Fragile X Associated Primary Ovarian Insufficiency” OR “Fragile X Premature Ovarian Failure” OR “FMR1 Related Primary Ovarian Insufficiency” OR “FMR1 Related Primary Ovarian Insufficiency” OR “Primary Ovarian Insufficiency, Fragile X Associated” OR “Primary Ovarian Insufficiency, Fragile X Associated” OR “Premature Ovarian Failure 1” OR “poor ovarian response”): ti, ab, kw  #2 (“Platelet-Rich Plasma” OR “Plasma, Platelet-Rich” OR “Platelet Rich Plasma”) : ti, ab, kw  #3 #1 AND #2 |
| CNKI(2) | ((主题=(卵巢早衰 + 家族性卵巢早衰 + 特发性卵巢早衰 + 早衰 + 卵巢功能早衰 + 早发性卵巢功能不全 + 早发性卵巢功能下降 + 卵巢储备下降 + 卵巢功能下降 + 卵巢低反应 + 卵巢反应不良)) AND 主题=富含血小板血浆) AND ((主题=(卵巢早衰 OR 家族性卵巢早衰 OR 特发性卵巢早衰 OR 早衰 OR 卵巢功能早衰 OR 早发性卵巢功能不全 OR 早发性卵巢功能下降 OR 卵巢储备下降 OR 卵巢功能下降 OR 卵巢低反应 OR 卵巢反应不良)) AND 主题=富含血小板血浆) |

Table S3. Characteristics of included studies

| Study | Area | Study design | diagnose | No. patients | | Age | | Number of cycles | | Duration | Primary outcome |
| --- | --- | --- | --- | --- | --- | --- | --- | --- | --- | --- | --- |
|  |  |  |  | Intervention | Control | Intervention | Control | Intervention | Control |  |  |
| Parvanov 2022 | Bulgaria | Prospective / Cohort | POR | 66 | 66 | 40.5(34–46) | 40.5(34–46) | NA | NA | NA | 2.4.7.8.10.11.17.18.19 |
| Aflatoonian 2021 | Iran | Prospective / Cohort | POR | 17 | 17 | 35.47±4.34 | 35.47±4.34 | NA | NA | September 2018 to February 2020 | 1.2.4.5,20.21 |
| Farimani 2021 | Iran | Retrospective / Cohort | POR | 96 | 96 | 38.30 ± 4.53 | 38.30 ± 4.53 | NA | NA | April 2018 to April 2020 | 1.2.4.5.7.10.12.26 |
| Navali 2022 | Iran | Prospective / Cohort | POR | 35 | 35 | 40.43±0.26 | 40.43±0.26 | NA | NA | April 2021 to May 2021 | 1.2.4.5.7.12.21 |
| Cakiroglu 2022 | Turkey | Prospective / Cohort | POR | 510 | 510 | 40.3±4.0 | 40.3±4.0 | NA | NA | January 2020 to December 2020 | 2.4.7.8.9.17.19.23.20.21.25.26 |
| Keikha 2022 | Iran | Prospective / Case Control | POR | 12 | 12 | 40.04±3.91 | 40.04±3.91 | 2.79±1.72 | 2.79±1.72 | August 2021 to December 2021 | 2.4.6.7.8.12.13 |
| Farimani 2019 | Iran | Prospective / Case Control | POR | 12 | 12 | 35.57±3.80 | 35.57±3.80 | 6.50±3.77 | 6.50±3.77 | NA | 7.12.21.26 |
| Sfakianoudis 2020 | Greece | Prospective / Case Control | POR | 18 | 12 | 35.11±1.57 | 35.92±1.93 | 10.06±2.62 | 10.17±4.76 | February 2017 to January 2019 | 2.4.5.8.10.13.14.15.17.20.22.25.26 |
| Tülek 2022 | Turkey | Retrospective / Cohort | POR | 50 | 50 | 38.1±4.4 | 38.1±4.4 | NA | NA | January 2018 and January 2021 | 1.3.6.7.10.17.20.25.26 |
| Pacu 2021 | Romania | Retrospective / Cohort | POR | 20 | 20 | 37.4±4.00 | 37.4±4.00 | NA | NA | February 2019 to February 2020 | 2.4.5.8.9.12.13.14.15.20.21.22.26 |

*Not applicable marked as NA*

1. E_2_ 2. FSH 3. P 4. AMH 5. LH 6. Endometrial thickness 7. Number of mature oocytes 8. Number of antral follicles 9. Number of oocytes taken 10. Number of metaphase II oocytes 11. Number of metaphase II oocytes 12. Number of excellent embryos 13. Duration of stimulation 14. Dose of Gonadotropin 15. Dose of estradiol trigger 16. Quality of metaphase II oocytes 17. Number of embryos on the fifth day 18. Quality of blastocysts 19. Number of blastocysts 20. Live birth rate 21. Natural pregnancy rate 22. Cancellation rate 23. Fertilization rate 24. Effective Rate 25.2 prokaryotic embryos 26. ART pregnancy rate

Table S4. Quality assessment of included studies

| Studies | Sample representativeness | Sample size | Response rate | Statistics reporting | Measurement tools |
| --- | --- | --- | --- | --- | --- |
| Parvanov 2022 | 1 | 0 | 1 | 1 | 1 |
| Aflatoonian 2021 | 1 | 0 | 1 | 1 | 1 |
| Farimani 2021 | 1 | 0 | 1 | 1 | 1 |
| Navali 2022 | 1 | 0 | 1 | 1 | 1 |
| Cakiroglu 2022 | 1 | 1 | 1 | 1 | 1 |
| Keikha 2022 | 1 | 0 | 1 | 1 | 1 |
| Farimani 2019 | 1 | 0 | 1 | 1 | 1 |
| Sfakianoudis 2020 | 1 | 0 | 1 | 1 | 1 |
| Tülek 2022 | 1 | 0 | 1 | 1 | 1 |
| Pacu 2021 | 0 | 0 | 1 | 1 | 1 |

1. Sample representativeness:

1 point: Population contained at least two age group spanning 10 years.

0 points: Population contained either one age group, or age span less than 10 years old.

1. Sample size:

1 point: Sample size was greater than or equal to 200 participants.

0 points: Sample size was less than 200 participants.

1. Response rate:

1 point: The response rate was above 80%.

0 points: The response rate was lower than 80%, or didn’t report a response rate.

1. Statistics reporting

1 point: The study reported descriptive statistics to describe the population (e.g., age, sex) with proper measures of dispersion (e.g., mean, standard deviation).

0 points: The study didn’t report descriptive statistics, incompletely reported descriptive statistics, or did not report measures of dispersion.

1. Measurement tools

1 point: The study adapted a validated measurement tool to determine the POR.

0 points: The study did not adapt a validated measurement tool to determine the POR.

Table S5 Results of PRP injection methods and ART selection

| Studies | ART method | Injection volume | Injection method | Times of Injection | Interval time | Observation node | Safety |
| --- | --- | --- | --- | --- | --- | --- | --- |
| Parvanov 2022 | IVF-ET(Decapeptyl 3.75mg, Gonal 300IU/day) | NA | Bilateral | 2-3 | 1 menstrual cycle | The third day of the treatment cycle | NA |
| Aflatoonian 2021 | Not promoting ovulation | 1.5 ml (first time)  3 ml (second time) | Unilateral | 1-2 | 3 months | the third day of menstruation | NA |
| Farimani 2021 | Not promoting ovulation | 2 ml | Unilateral | 1-2 | NA | the 2nd to 4th day of menstruation | NA |
| Navali 2022 | Letrozole (5mg), rhFSH (225 IU), hMG (75 mg/kg), hCG (10000 IU) | 2 ml | Bilateral | 1-2 | 2 months or 3 menstrual cycle | the day of injection of hCG | Not seen |
| Cakiroglu 2022 | rhFSH (300IU), hCG (10000 IU) | NA | Unilateral /Bilateral | 2 | 6 weeks | the 2nd to 4th day of menstruation | NA |
| Keikha 2022 | GNRH antagonist (150IU), HMG (150IU), Ketopeptide (25mg)  hCG (10000 IU) | 4 ml | Unilateral | 2 | 70 days | 36 hours after injection of hCG | NA |
| Farimani 2019 | NA | 2 ml | Bilateral | 1-2 | 2-3 months | the 2nd to 4th day of menstruation | Not seen |
| Sfakianoudis 2020 | intracytoplasmic sperm injection | 4 ml (Platelet concentration ＞100,000) | Bilateral | 1 | NA | 3 months | Not seen |
| Tülek 2022 | COH, intracytoplasmic sperm injection | 2 ml | Bilateral | 1 | NA | 6 months | NA |
| Pacu 2021 | NA | 2-4 ml (Platelet concentration 250,000-85,000) | Bilateral | 1 | NA | 6 months | Not seen |
